# Fulranumab in patients with interstitial cystitis/bladder pain syndrome: observations from a randomized, double-blind, placebo-controlled study

**DOI:** 10.1186/s12894-016-0193-z

**Published:** 2017-01-05

**Authors:** Hao Wang, Lucille J. Russell, Kathleen M. Kelly, Steven Wang, John Thipphawong

**Affiliations:** 1 Office of Translational Research, National Institute of Neurological Disorders and Stroke (NINDS), Bethesda, MD USA; 2 Janssen Research & Development, LLC, Raritan/Titusville, NJ USA

**Keywords:** Fulranumab, Pain, Analgesia, Interstitial cystitis, Bladder

## Abstract

**Background:**

This study was designed to evaluate the efficacy and safety of fulranumab, a fully human monoclonal antibody directed against nerve growth factor (NGF), for pain relief in patients with interstitial cystitis/bladder pain syndrome (IC/BPS).

**Methods:**

In this multicenter, double-blind study, adults with IC/BPS (i.e., interstitial cystitis symptom index [ICSI] total score ≥8) accompanied by chronic, moderate-to-severe pain were randomized to fulranumab 9 mg or matching placebo, administered subcutaneously at weeks 1, 5, and 9. The primary efficacy endpoint was change from baseline to study endpoint (week 12 or at withdrawal) in average daily pain intensity score. Key secondary endpoints included change from baseline to study endpoint in worst pain intensity score, ICSI total score, Pelvic Pain and Urgency/Frequency total score, Patient Perception of Bladder Condition score, and global response assessment.

**Results:**

This study was terminated prematurely based on concern that this class may be associated with rapidly progressing osteoarthritis or osteonecrosis. Thirty-one patients (of the targeted 70 patients) were randomized, 17 to placebo and 14 to fulranumab, with 15 and 10 patients, respectively, receiving all 3 doses of double-blind treatment. In ANOVA analyses, there was no statistically significant difference between treatment groups for the primary endpoint (LS mean difference [95% CI] vs. placebo, −0.2 [−1.52, 1.10]) or any of the secondary endpoints. Fulranumab was well tolerated, with no patient discontinuing due to an adverse event or experiencing a joint-related serious adverse event over a 26-week follow-up period. No events related to the neurologic or motor systems were reported.

**Conclusions:**

Efficacy was not demonstrated in the present study with the single dose tested and a limited sample size, leading to lack of statistical power. These findings do not exclude the possibility that fulranumab would provide clinical benefit in a larger study and/or specific populations (phenotypes) in this difficult to treat pain condition.

**Trial registration:**

NCT01060254, registered January 29, 2010.

## Background

Pain (suprapubic related to bladder filling, pelvic, and/or in extragenital locations such as the lower abdomen/back) is the hallmark symptom of interstitial cystitis/bladder pain syndrome (IC/BPS) [1]. Several lines of evidence suggest that IC/BPS can be conceptualized as a bladder pain syndrome associated with not only chronic pain, but also voiding-related symptoms and, in many, other chronic systemic pain disorders. As such, pain management is central to multimodal therapeutic strategies for IC/BPS, given its bearing on psychosocial functioning and quality of life [2, 3]. In fact, recently published IC/BPS guidelines from the American Urological Association recommend pain management as first-line treatment, which should be offered to all patients [1]. Despite the need of IC/BPS patients for analgesia, effective pharmacological treatment for their chronic pain remains elusive [4–6].

In the midst of a growing need for alternative analgesics, nerve growth factor (NGF) antagonism is a focus of drug research and development, with anti-NGF agents in the spotlight [7]. Experimental and clinical evidence has established the major role of NGF in the generation and maintenance of pain states, and specifically in bladder pain in animal models [7–9]. Several humanized and fully human anti-NGF monoclonal antibodies have entered clinical trials as potential analgesics, with preliminary results showing significant improvement in chronic pain from osteoarthritis [10] and diabetic peripheral neuropathy [11, 12] and mixed results for relief of chronic low back pain [13]. Efficacy for interstitial cystitis was also tested initially with positive results [14], but failed to show treatment effect versus placebo in a second study of chronic prostatitis/chronic pelvic pain syndrome [15].

Fulranumab, a fully human recombinant monoclonal antibody (immunoglobulin G), is a potent inhibitor of human NGF. Results from phase 2, randomized, placebo-controlled studies of fulranumab demonstrate a positive dose response in diabetic peripheral neuropathic pain [11], mixed efficacy results versus placebo and statistically significant improvement versus an opioid in pain of osteoarthritis [16, 17], and did not separate from placebo for low-back pain [18]. We herein report the results of a phase 2a study conducted to explore the efficacy and safety profile of fulranumab, as compared to placebo, in patients with moderate-to-severe chronic bladder pain from IC/BPS. On December 23, 2010, the United States Food and Drug Administration (FDA) placed ongoing fulranumab studies on clinical hold because of a concern that the entire class of anti-NGF antibodies may be associated with a condition representing either rapidly progressing osteoarthritis or osteonecrosis [19]. As a result, the sponsor discontinued this study prematurely, after having enrolled only 31 of the targeted 70 patients.

## Methods

### Ethical practices

An Independent Review Board (US) or Research Ethics Board (Canada) at each study site approved the study protocol and protocol amendments. The study was conducted in accordance with the ethical principles that have their origin in the Declaration of Helsinki, consistent with Good Clinical Practices and applicable regulatory requirements. All patients provided written informed consent before study participation. The study is registered at clinicaltrials.gov, NCT 01060254.

### Patients

Study participants were adult (≥18 to 80 years, inclusive) men and women with IC/BPS based on a total score ≥ 8 on the validated O’Leary-Sant interstitial cystitis symptom index (ICSI) [20, 21] and chronic bladder pain for at least 6 months prior to screening, accompanied by urinary urgency, urinary frequency (≥8 voids daily), and/or nocturia. On the basis of patients’ self-assessment of pain using a 11-point numerical rating scale (NRS) (0 = “no pain”, 10 = “worst pain imaginable”), the mean of the average pain intensity scores for the last 7 days of screening had to be ≥5 based on at least 6 of 7 days for eligible patients [22]. Other key eligibility criteria required that patients had no evidence of a urinary tract infection or significant urological disease, including neurogenic bladder and diabetic cystopathy, and had not received intravesical therapy or undergone cystoscopy during the 6 weeks prior to screening. In addition, they had not received opioid analgesic in a dosage of oral morphine equivalent ≥ 40 mg/day or changed drugs known to affect IC/BPS-associated pain (i.e., antidepressants, antihistamines, antispasmodics, anticholinergics, anticonvulsants) within the 4 weeks before screening.

### Study design

This randomized, double-blind, placebo-controlled, multicenter study was conducted between March 2010 and June 2011 (last study visit) at 12 study sites (10 in US and 2 in Canada). Reports of spontaneous reporting of joint replacements were collected through November 2011 (explanation provided under *Safety Assessments*). The study comprised a screening period of up to 4 weeks, a 12-week double-blind treatment period, and a post-treatment period that concluded 26 weeks after the final dose of study drug was administered.

Eligible patients were randomized in a 1:1 ratio, based on a computer-generated randomization schedule, to fulranumab 9 mg or matching placebo injected subcutaneously (SC) into the thigh (or abdominal wall, only if the thigh was not feasible) once every 4 weeks (i.e., at weeks 1, 5, and 9 visits). Randomization was balanced using randomly permuted blocks and was stratified by the presence versus absence of glomerulations and/or Hunner’s ulcer on cystoscopy [23] and by baseline body weight (<85 kg vs. ≥ 85 kg). Patients were followed during the double-blind treatment period at clinic visits conducted at baseline and every other week through week 13.

If patients used regularly scheduled bladder pain medications within 3 months before screening, they could continue taking them (with no dose changes) concomitantly with study drug. In addition, rescue use of acetaminophen (up to 3000 mg/day) was allowed throughout the study.

#### Efficacy assessments

At each study visit, patients received a diary into which they recorded a bladder pain NRS score for average and worst pain over the previous 24 h, urinary frequency (daytime and nocturnal), and interference with sleep for the 7 days before clinic visits. At all clinic visits, patients rated the degree to which their IC/BPS caused them problems using the Patient Perception of Bladder Condition (PPBC) instrument [24] and the number of nocturnal awakenings for voiding, pain with sexual activity, bladder/pelvic pain, and the frequency of bother with these symptoms using the Pelvic Pain and Urgency/Frequency Questionnaire (PUF) [25]. At selected clinic visits (weeks 5, 9, and 13), patients also rated the presence and extent of their IC/BPS symptoms (urinary urgency, urinary frequency, nocturia, pain/burning in the bladder) by completing an ICSI questionnaire and their overall status since beginning study medication by completing the 7-point global response assessment (GRA): very much worse; much worse; minimally worse; not changed; minimally improved; much improved; and very much improved.

#### Safety assessments

Investigators monitored adverse events throughout the study. They also performed other standard safety assessments (i.e., clinical laboratory tests, vital signs, physical examination) at prespecified time points.

An independent data monitoring committee, including 3 medical experts in pain and neurology and 1 statistician, reviewed unblinded safety data on an ongoing basis to ensure patient safety. This review did not identify any safety signal that met prespecified stopping rules.

In response to an FDA request, additional information to assess patient’s joints was collected to evaluate whether rapidly progressing osteoarthritis or osteonecrosis occurred during the study. The tests included imaging data and/or historical data (e.g., X-rays, MRIs, ultrasounds, historical data pertaining to the joint replacement) of any joint that had been replaced or in which a relevant joint-related adverse event occurred. The study remained open until November 2011 to accommodate this request.

### Data analysis

Efficacy analyses were performed on the intent-to-treat (ITT) data set, namely, all patients who were randomized, received at least 1 dose of study drug, and had at least 1 efficacy evaluation during the double-blind treatment period.

#### Efficacy endpoints and analyses

The primary efficacy endpoint, defined as change in average pain intensity from the baseline bladder pain intensity score at study endpoint (i.e., end of the double-blind treatment phase or at the withdrawal visit), was analyzed using an analysis of variance (ANOVA) model, which included treatment group as factor. For patients who terminated before completing 12 weeks of treatment, the last observed data were carried forward to endpoint for analysis. Analyses of secondary efficacy endpoints were conducted using a similar ANOVA model.

#### Sample size determination

In the absence of data on bladder pain effect size in IC/BPS patients and specific effects of fulranumab, sample size assumptions for this study were based on those from another study [16] that was ongoing at the time the protocol for this study was written. Assuming a treatment difference of 1.4 in the change from baseline in average pain intensity score between fulranumab and placebo, a standard deviation (SD) of 2.4, and an overall discontinuation rate of 20%, then a sample size of 35 patients per treatment group or total 70 patients in the entire study would have provided 78.8% power using a type 1 error rate of 0.1 and a 1-sided 2 sample t-test.

## Results

### Patients

A total of 83 patients were screened and 31 eligible patients were randomized, 17 to placebo and 14 to fulranumab. On December 23, 2010, the FDA placed ongoing fulranumab studies on clinical hold because of a concern that the entire class of anti-NGF antibodies may be associated with a condition representing either rapidly progressing osteoarthritis or osteonecrosis [19]. As a result, the sponsor discontinued this study prematurely, after having enrolled only 31 of the targeted 70 patients.

Of the 31 randomized patients, 24 completed the study and 7 were withdrawn from the study prematurely (Table 1). Two of 17 patients in the placebo group and 4 of 14 patients in the fulranumab group did not receive all 3 doses of double-blind study drug.

**Table 1:** Patient disposition

	Number (%) of patients	
	Placebo	Fulranumab 9 mg
Randomized	17 (100)	14 (100)
Received all 3 doses of study drug	15 (88)	10 (71)
Completed the study	14 (82)	10 (71)
Withdrew from the study	3 (18)	4 (29)
Sponsor discontinued the study	1 (6)	0 (0)
Patient withdrew consent	2 (12)	0 (0)
Other	0 (0)	4^a^ (29)

^a^Two patients did not meet inclusion/exclusion criteria, 1 patient missed the Week 9 dose, and 1 patient withdrew due to the clinical hold

The treatment groups were well-matched based on demographic and most other baseline characteristics (Table 2). The ITT data set was composed primarily of women (83.9%); the mean (SD) age was 48.2 (12.34) years. Patients in the placebo group reported longer IC/BPS disease duration (mean, 9.4 vs. 5.8 years for the fulranumab group) and higher daytime urinary frequency (mean, 16.5 vs. 10.8); mean baseline daily NRS scores for average (6.6 vs. 5.7) and worst (7.6 vs. 6.6) bladder pain scores were similar between the treatment groups, as was the frequency of nocturnal urinary frequency (4.9 vs. 3.7). Of the UPOINT domains, the fulranumab group had a higher percentage of patients in the infection and neurological/systemic domains than the placebo group (Table 2).

**Table 2:** Demographic and baseline characteristics (ITT data set)

Characteristic	Placebo (*N* = 17)	Fulranumab 9 mg (*N* = 14)
Sex, n (%)		
Female	13 (76.5)	13 (92.2)
Male	4 (23.5)	1 (7.1)
Race, n (%)		
White	16 (94.1)	13 (92.9)
Black or African American	1 (5.9)	1 (7.1)
Age (years)		
Mean ± SD	46.2 (13.56)	50.6 (10.68)
Range	25–69	38–78
Body weight (kg)		
Mean ± SD	78.9 (16.61)	71.7 (26.12)
Range	54–117	46–148
UPOINT clinical phenotyping, n (%)		
Urinary	17 (100)	14 (100)
Psychosocial	5 (29)	3 (21)
Organ-specific	16 (94)	11 (79)
Infection	4 (24)	5 (36)
Neurological/systemic	7 (41)	9 (64)
Tender point	12 (71)	9 (64)
Duration of interstitial cystitis (years)		
Mean ± SD	9.4 (9.87)	5.8 (5.44)
Range	2–40	1–20
Hunner’s ulcer and/or glomerulation, n (%)		
Yes	9 (52.9)	10 (71.4)
No	8 (47.1)	4 (28.6)

Sixteen patients in the placebo group and 12 of 14 (86%) in the fulranumab group received concomitant IC/BPS medications with study drug, the most common being pentosan polysulfate sodium (PPS) (7 patients in each treatment group); a benzodiazepine (6 and 5 patients in the placebo and fulranumab groups, respectively), a corticosteroid (4 and 2 patients in the respective groups), an antidepressant (7 and 3 patients in the respective groups), an antihistamine (5 and 6 patients in the respective groups), an anticholinergic (2 and 3 patients in the respective groups), an antispasmodic (1 and 3 patients in the respective groups), and an anti-infective agent (3 and 2 patients in the respective groups).

### Efficacy results

**Fig. 1:**
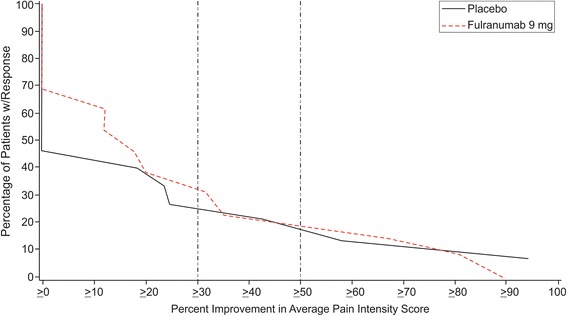
Distribution of Pain Responder Rates at the Study Endpoint

There was no statistically significant difference between fulranumab and placebo for the primary efficacy endpoint (Table 3). The responder rates based on 30 and 50% improvement at the study endpoint in the average pain intensity score were also similar between the treatment groups (Fig. 1). The responder rate based on 30% improvement in average pain score at study endpoint in the ITT data set was 30.8% for patients treated with fulranumab and 33.3% for placebo patients.

**Table 3:** Summary of efficacy at end of double-blind treatment (ITT Data Set)

	Placebo (*N* = 17)	Fulranumab 9 mg (*N* = 14)
Primary Endpoint – Average Pain Intensity Score^a^		
Baseline, mean ± SD	6.8 ± 1.30	6.1 ± 1.11
Change at end of double-blind treatment period, mean ± SD	−1.2 ± 1.88	−1.4 ± 1.41
Difference of means (95% CI)		−0.2 ± 0.64 (−1.52, 1.10)
% Responders defined as:		
≥ 30% improvement in average pain intensity score^a^	5 (33.3%)	4 (30.8%)
≥ 50% improvement in average pain intensity score^a^	3 (20.0%)	3 (23.1%)
Secondary Endpoint – Worst Pain Intensity Score^a^		
Baseline, mean ± SD	7.6 ± 1.32	6.9 ± 1.13
Change at end of double-blind treatment period, mean ± SD	−1.5 ± 1.90	−1.5 ± 1.55
Difference of means (95% CI)		−0.1 ± 0.66 (−1.44, 1.28)
Other Secondary Endpoints:		
ICSI Total Score^b^		
Baseline, mean ± SD	16.5 ± 2.92	14.6 ± 3.27
Change at end of double-blind treatment period, mean ± SD	−3.0 ± 3.97	−4.4 ± 4.40
Difference of means (95% CI)		−1.4 ± 1.50 (−4.50, 1.65)
PUF Total Score^c^		
Baseline, mean ± SD	26.2 ± 6.86	24.3 ± 4.89
Change at end of double-blind treatment period, mean ± SD	−4.2 ± 5.38	−6.1 ± 5.16
Difference of means (95% CI)		−1.9 ± 1.91 (−5.81, 1.99)
PPBC Score^d^		
Baseline, mean ± SD	4.9 ± 0.86	4.7 ± 0.61
Change at end of double-blind treatment period, mean ± SD	−0.5 ± 1.12	−0.9 ± 1.23
Difference of means (95% CI)		−0.3 ± 0.42 (−1.19, 0.54)
Daytime urinary frequency^e^		
Baseline, mean ± SD	16.5 ± 11.66	11.0 ± 6.68
Change at end of double-blind treatment period, mean ± SD	0.8 ± 3.92	0.5 ± 2.95
Difference of means (95% CI)		−0.3 ± 1.33 (−3.02, 2.44)
Nocturnal urinary frequency^e^		
Baseline, mean ± SD	5.0 ± 4.36	3.8 ± 3.28
Change at end of double-blind treatment period, mean ± SD	0.6 ± 5.33	−0.4 ± 1.33
Difference of means (95% CI)		−1.0 ± 1.52 (−4.16, 2.09)

*ICSI* O’Leary-Sant Interstitial Cystitis Symptoms Index, *PPBC* Patient Perception of Bladder Condition, *PUF* Pelvic Pain and Urgency/Frequency Questionnaire

^a^An 11-point scale, ranging from 0 (“no pain”) to 10 (“pain as bad as you can imagine”)

^b^Sum of 4 individual question ratings, each on a 0 to 5 scale, where higher scores indicate worse symptoms

^c^A 12-item scale, with total score (sum of the symptom and bother subscale scores) ranging for 0 to 35; higher scores indicate worse symptoms

^d^Single item global measure of bladder condition rated on a 6-point scale

^e^Derived as an average number of events over the 7 consecutive days prior to each visit

Mean of all non-missing scores after first dose and up to last non-missing score for the 12-week double-blind period

There were no statistically significant between-group differences for any of the secondary endpoints (Table 3), however, there was a trend of better GRA. Five (35.7%) patients in the fulranumab group and 4 (23.5%) patients in the placebo group reported a GRA score of “very much improved” or “much improved”, 5 patients in each group (35.7% and 29.4%, respectively) reported a GRA score of “minimally improved”, and 4 (28.5%) and 8 (47.1%) patients in the respective groups reported “not changed”, “minimally changed”, or “much worse”.

### Safety results

The safety profile of fulranumab was similar to that of placebo. The most frequently reported adverse events in the fulranumab group were diarrhea, carpal tunnel syndrome, and urinary tract infection (Table 4). In both treatment groups, most adverse events were mild or moderate in intensity and were reported to be either not related or doubtfully related to the study drug. The only serious adverse event (kidney infection) was reported in the placebo group. No study patient discontinued study drug due to an adverse event.

**Table 4:** Treatment-emergent adverse events reported in ≥10% of patients in either treatment group

	Placebo (*N* = 17)	Fulranumab 9 mg (*N* = 14)
Any adverse event	11 (65%)	8 (57%)
Diarrhea	2 (12)	3 (21)
Carpal tunnel syndrome	0	2 (14)
Urinary tract infection	2 (12)	2 (14)
Pain	2 (12)	0
Pain in extremity	2 (12)	0
Paresthesia	2 (12)	0
Sinusitis	2 (12)	0

Adverse events are presented in decreasing order of incidence for the fulranumab group

Regarding adverse events of clinical interest (related to anti-NGF antibodies), none of the study patients had joint-related serious adverse events. No event related to bradycardia, hepatic failure, acute renal failure, or neurologic and motor system was reported. One patient (in the placebo group) experienced a hypotension-related adverse event.

## Discussion

In this multicenter, double-blind, randomized study of 31 adults with a diagnosis of IC/BPS who suffered from chronic, moderate-to-severe bladder pain, a treatment effect (vs. placebo) was not observed with fulranumab, based on the primary study endpoint or any of the secondary endpoints. A trend for improvement was observed on the GRA, considering all subcategories, however this finding needs to be confirmed. Patient enrollment was curtailed at an early stage due to a regulatory hold on clinical studies, preventing complete efficacy and safety analysis, reducing power (from 79 to 52%), and limiting conclusions. In addition, an imbalance in the baseline characteristics of the two treatment groups was detected, further diminishing the ability to definitively rule out a potential treatment effect. The development of anti-NGF antibodies was re-initiated in 2015 in patients with osteoarthritis and low back pain (NCT02301234, NCT0252818, NCT02528253, and NCT02683239; at clinical trials.gov). Further development of anti-NGF agents in patients with IC/BPS will be dependent on the safety profile observed in the osteoarthritis and low back pain studies.

The therapy of IC/PBS is usually multimodal and is rarely satisfactory [26–28]. With the exception of PPS (Elmiron), there are currently no approved pharmacologic therapies in the United States for IC/BPS. Many pharmacologic therapies such as antidepressants and antihistamines are used off label. Other therapies include intravesical installation of agents, bladder distention, and surgical intervention [26–28]. Promising agents have been studied, but with mixed results, and none have gained regulatory approval. In this regard, Evans et al. reported significant improvement in pain and the GRA 6 weeks after a single IV infusion of another anti-NGF monoclonal antibody, but no improvement on the ICSI in a pilot study of 64 patients with IC/BPS [14]. The same anti-NGF antibody was tested in a second clinical study of over 200 patients with IC/BPS that was terminated for futility (NCT00999518) [29].

Limitations of this study were early termination of the study resulting in small sample size and some patients not receiving 3 doses of study drug, as well as imbalance between the treatment groups based on baseline characteristics. For example, patients in the placebo group reported longer IC/BPS disease duration (mean, 9.4 vs. 5.8 years for the fulranumab group) and higher daytime urinary frequency (mean, 16.5 vs. 10.8); and of the UPOINT domains, the fulranumab group had a higher percentage of patients in the infection and neurological/systemic domains than the placebo group (Table 2). In addition, due to the heterogeneous population, multiple factors that are impractical to stratify may confound or increase the noise of the study; these include concomitant IC/BPS medication types/rate of usage, and commonly comorbid conditions (e.g., irritable bowel disease, depression, pelvic floor dysfunctional disease) that define different clinical phenotypes [30].

Clinical trials of IC/BPS have been limited, in general, by substantial heterogeneity in methodology, symptoms assessment, duration of treatment, and follow-up [31]. For example, although initial trials were positive for PPS [32, 33], subsequent large confirmatory trials have had mixed results [34]. Experts have recently advanced the use of clinical phenotypes based on UPOINT or pain mapping/pain location in IC/BPS research [30, 35]. With regard to analgesia for IC/BPS, future studies should include mapping of pain sites (i.e., restricted to the pelvis; pain sites beyond the pelvis) and the quality/severity of pain at those sites, assuring differentiation between IC/BPS and other pain syndromes by patients, as well as correlate findings from these assessments with associated comorbid, chronic pain conditions [30]. While this study was designed to collect data pertaining to the different clinical phenotypes using the UPOINT system and conduct exploratory subgroup analyses, these analyses were not feasible due to early study termination and the reduced sample size. Future studies of adequate sample size should further explore treatment response according to clinical phenotypes.

## Conclusions

In patients with moderate to severe chronic bladder pain from IC/BPS, fulranumab at the single dose (9 mg) tested failed to show analgesic activity as compared with placebo, although the study findings are limited by early study termination and imbalance in the baseline characteristics of the study population. IC/BPS remains a difficult medical condition in need of satisfactory therapies.

## Abbreviations

ANOVA: Analysis of variance
FDA: Food and Drug Administration
GRA: Global response assessment
IC/BPS: Interstitial cystitis/bladder pain syndrome
ICSI: Interstitial cystitis symptom index
ITT: Intent-to-treat
NGF: Nerve growth factor
NRS: Numerical rating scale
PPBC: Patient Perception of Bladder Condition
PPS: Pentosan polysulfate sodium
PUF: Pelvic Pain and Urgency/Frequency Questionnaire
SC: Subcutaneously
SD: Standard deviation
